# Annotations

**Published:** 1888-04-21

**Authors:** 


### Annotations.

**Lying-in Hospitals.**

Mr. Thomas Ryan's paper, read a few weeks ago at The Hospitals Association, appears to have caused considerable feeling in certain quarters. A controversy, started in the *Medical Press and Circular* immediately after the reading of the paper, is still continuing. Mr. Ryan at the outset took care to entrench himself behind a solid rampart of facts. Facts, in the world of physical science at any rate, are considered to be arguments which admit of no refutation. We are not aware that anybody out of France, when told that the facts were against him, has ventured to declare—"So much the worse for the facts." The truth seems to be that one particular lying-in-hospital, which we refrain from naming, has lagged somewhat behind other metropolitan institutions of a like kind, at any rate in its results. The assumption is that it has lagged behind them either in its choice of methods or in the thoroughness of their application, or in both. That it really has been left considerably in the rear in its rate of mortality seems to be indisputable. Mr. Ryan not only gives a statistical table, the accuracy of which is not questioned, but adds a variety of details, all bearing on the one question of mortality, and all tending to show that the adoption and thorough practice of the antiseptic system of hospital management results in a lessened mortality, which is as marked as it is gratifying. Of the soundness of Mr. Ryan's conclusions on this head there cannot be the least shadow of doubt. The evidence from general and special hospitals, the experience of operating surgeons and general practitioners, the accumulated facts gathered from all quarters are of so conclusive a nature that nothing but prejudice or stupidity could continue tò doubt. The essential principle of Listerism is cleanliness—that we readily admit. But cleanliness is a condition which requires all kinds of care and effort for its maintenance, and sometimes it is the unwillingness to take trouble which constitutes the atmosphere of disease. The controversy can only be productive of good. There is evidence to show that within the last twelve months the hospital in question has experienced a greatly lowered rate of mortality, a rate which compares favourably with the best results of its most successful competitors. This fact we are glad to announce. No one need question that the physicians and managers of all hospitals are anxious to do the utmost which is possible in order to effect the very best results. They would be monsters if they wished otherwise. But it cannot be expected that every new method, however good in itself, will appeal with the same convincing force to every mind, and always ensure for itself a simultaneous and universal adoption. Mr. Ryan's paper has been very valuable, as showing the dangers of past methods and conditions, and equally valuable as tending to make manifest that the adoption of better methods is now universal, and that the results too are becoming more uniformly excellent than at any previous time.

**Total Abstinence and Poor Relief.**

Those who are familiar with a certain book on "Iceland Snakes" will remember the writer's opening sentence—"There are *no* snakes in Iceland." It would seem that there is exactly the same connexion between total abstainers and poor relief. According to the testimony of those who ought to know, total abstainers practically never require "poor relief." That is a great word to say; but it is not said without book. The Bishop of London presided on Saturday week at a conference in Exeter Hall, convened by the National Temperance League, of which he is president. The conference consisted exclusively of workhouse masters and relieving officers associated with metropolitan unions. Ten or twelve of those experienced officials took part in the debate, and expressed the decided conviction that a great part of the poverty and consequent suffering of the poor is caused by their intemperate habits. This is the conviction iterated and reiterated again and again by all those who know the habits of the working-classes. Strong drink is their ruin. Mr. Hunt, the relieving officer of Marylebone, wrote: "If it were not for intemperance I should soon have to join the unemployed." From a medical point of view, strong drink is as great a curse as it is from the social and economic standpoint. Not only relieving officers, but thousands of doctors would have to join the unemployed if it were not for the drinking habits of the people. We have sometimes been censured by correspondents for what they considered our illogical position in not declaring ourselves total abstainers. But we think that a man who, although he refuses to be deprived of his liberty of choice, nevertheless habitually leads a practically abstinent life, is more likely to be listened to by non-abstainers than one who by his conduct shows that he is determined to see only one side of the question. Be that as it may, no one can be more convinced than the present writer that intemperance, however slight, is an evil of the most shameful and injurious character. Almost any steps except those which inflict positive injury upon others are justifiable in the diminishing of the enormous injuries and dangers of the drink traffic. No persuasion can be too urgent, no measures too stern, short of wrong-doing, which will enable the people to free themselves from the incubus which weighs them down to their destruction. Apparently they have neither the sense nor the strength of mind to effect their own salvation. All good men, abstainers and non-abstainers, should unite both heart and brain in order to bring about a better state of things for those whose lives, always hard enough, are made tenfold more wretched by their own mad self-indulgence.

**Women and Faddists.**

Mr. Walter Besant is anxious to reduce women to the level of domestic hangers-on. They are not to be permitted even to understand that life is a struggle and demands both courage and sense. The International Conference of Women, which has just been in session at Washington, does not agree with the popular novelist. As the result of the conference, a permanent International Association has been formed, with Mrs. Fawcett as its president, which consists of women who are resolved to help other women to help themselves. The average educated English, or rather European woman, has a mental capacity superior to that of the average male African, or even the more civilised Hindoo. The men of those countries are not nursed in a lap of indulgence by Mother Nature, but must strive or starve. The women who have placed themselves under Mrs. Fawcett's leadership do not seem to be of the weak mould which whines and entreats instead of bestirring itself and putting its shoulder to the wheel. They have the intelligence to look difficulties in the face, and the courage to face them boldly. Certain facts were made known during the sittings of the Conference which will act as powerful incentives to steady and hopeful effort in the future. It seems that among civilised races women now find paid employment in nearly three hundred different callings, whereas so recently as 1840 they had only entered upon seven such occupations. It has often been said that women are imitators, but cannot originate. This is proved to be a popular delusion. Up to 1886 no fewer than 1,935 patents for new inventions had been taken out by women; and these included, among other things, fire escapes, steam generators, cow-milkers, churns, beehives, and the first submarine telescope. In New York, 200,000 women now find 100 different occupations open to them, as against twenty a generation ago. When women, without special co-operation, can reach the standing ground already attained, it is idle to talk of their incapacity or their want of pluck. Now that they have united for counsel and mutual guidance much more rapid advancement may be expected.

